# Comment on ‘Oxidative Stress‐Induced Expression Levels of PXDN and NF‐κB in Type 2 Diabetic Patients With Nephropathy’

**DOI:** 10.1002/edm2.70284

**Published:** 2026-07-12

**Authors:** Rofia Ali, Muhammad Faizan Khan

**Affiliations:** ^1^ University of Health Sciences Lahore Pakistan; ^2^ Nowshera Medical College Nowshera Pakistan; ^3^ Khyber Medical University Peshawar Pakistan

To the Editor,

1

We read with keen interest the article by Hanin et al. [1] discussing the potential role of peroxidasin (PXDN) gene and Nuclear factor‐kappaB (NF‐κB) levels as an early biomarker for diabetic nephropathy in Type 2 diabetes mellitus patients. The article is of great importance as it aims to identify a novel, reliable diagnostic biomarker for diabetic nephropathy. Diabetic nephropathy remains a leading cause of chronic renal disease, imposing an immense clinical and socioeconomic burden by significantly increasing the chances of cardiovascular mortality. Conventional diagnostic markers including creatinine and serum albumin appear only after substantial structural damage to the renal parenchyma has occurred. The article also highlighted a new association between oxidative stress and levels of peroxidasin and Nuclear factor‐κB. The author's approach to search for a minimally invasive tool to arrest the progression of diabetic nephropathy prior to irreversible tissue damage is highly commendable. A stringent appraisal of the study design and methodological framework reveals critical limitations that significantly constrain the implications of these findings for clinical practise.

Firstly, there is a clear mismatch between the study's title and its findings; the title and abstract frame PXDN expression levels as ‘oxidative stress‐induced’. The key markers for oxidative stress discussed in the study are malondialdehyde (MDA), Total Antioxidant Capacity (TAC), and Nuclear factor‐κB (NF‐κB). Comparison of malondialdehyde (MDA) and Total Antioxidant Capacity (TAC) among the studied groups shows a positive correlation with oxidative stress, but there was no significant correlation with PXDN gene expression levels. This creates an unaddressed gap between the literature‐derived hypothesis and the study's actual findings. Furthermore, the hypothesis‐ ‘oxidative‐stress induced’ expression of PXDN‐ was nullified by another finding in the study showing no correlation between Nuclear factor‐κB (NF‐κB) and PXDN expression. Additionally, the study establishes the characteristic PXDN gene expression in renal tissue. Still, previous studies confirm the presence of PXDN genes not only in renal tissue but also in the heart and vascular endothelium [2, 3]. The study identifies NF‐κB as a main contributor to the pathogenesis of diabetic nephropathy, but evidence synthesized by Lawrence et al. [4] indicates that NF‐κB is a ubiquitous regulator of inflammation and cannot be definitively localized to a renal source.

Secondly, there is a major statistical setback in how the PXDN diagnostic cutoff was calculated. The authors came up with a cut‐off value of > 5.4 fold‐change without explaining that the selection was based upon the ‘Youden's index’ or ‘closest‐to‐top‐left’ [5]. The AUC score was calculated using the same dataset of 60 patients; in statistics, this is called the re‐substitution bias. STARD guidelines clearly state that you cannot test a diagnostic rule on the same dataset used to build it [6]. Another concerning finding from the study is the sensitivity of 65%, which is extremely low for a screening test meant to diagnose a disease early.

Finally, the genetic testing protocols have two loopholes. First, the authors omitted the core details like primer sequences and amplification efficiency, which means this study fails to comply with both the original 2009 rules and the latest MIQE 2.0 standards from 2025 [7]. Without these metrics, independent replication is impossible, and the final data cannot be mathematically verified. Furthermore, using the Glyceraldehyde 3‐phosphate dehydrogenase (GAPDH) gene as the only baseline control is a fatal flaw in a diabetes study, since high blood sugar suppresses its expression by 66% [8]. This drop in your baseline gene elevates the apparent expression of the target gene.

In conclusion, while investigating PXDN is a promising line of research, the lack of mechanistic correlation, the absence of independent validation, and the flawed PCR normalization weaken the study's definitive claims. Before PXDN can be considered a clinically useful biomarker, these preliminary findings must be rigorously verified using MIQE 2.0‐compliant protocols in a larger, independent, long‐term study.

## Author Contributions

**Rofia Ali:** conceptualization, investigation, writing – original draft, methodology, validation, visualization, writing – review and editing, resources. **Muhammad Faizan Khan:** writing – review and editing, conceptualization, supervision.

## Funding

The authors have nothing to report.

## Ethics Statement

The authors have nothing to report.

## Consent

The authors have nothing to report.

## Conflicts of Interest

The authors declare no conflicts of interest.

## Data Availability

Data sharing not applicable to this article as no datasets were generated or analysed during the current study.
